# The Effects of Technological Developments on Work and Their Implications for Continuous Vocational Education and Training: A Systematic Review

**DOI:** 10.3389/fpsyg.2020.00918

**Published:** 2020-05-08

**Authors:** Patrick Beer, Regina H. Mulder

**Affiliations:** Faculty of Human Sciences, University of Regensburg, Regensburg, Germany

**Keywords:** technology, work characteristics, continuous vocational education and training, automation, work demands, systematic review

## Abstract

Technology is changing the way organizations and their employees need to accomplish their work. Empirical evidence on this topic is scarce. The aim of this study is to provide an overview of the effects of technological developments on work characteristics and to derive the implications for work demands and continuous vocational education and training (CVET). The following research questions are answered: What are the effects of new technologies on work characteristics? What are the implications thereof for continuous vocational education and training? Technologies, defined as digital, electrical or mechanical tools that affect the accomplishment of work tasks, are considered in various disciplines, such as sociology or psychology. A theoretical framework based on theories from these disciplines (e.g., upskilling, task-based approach) was developed and statements on the relationships between technology and work characteristics, such as complexity, autonomy, or meaningfulness, were derived. A systematic literature review was conducted by searching databases from the fields of psychology, sociology, economics and educational science. Twenty-one studies met the inclusion criteria. Empirical evidence was extracted and its implications for work demands and CVET were derived by using a model that illustrates the components of learning environments. Evidence indicates an increase in complexity and mental work, especially while working with automated systems and robots. Manual work is reported to decrease on many occasions. Workload and workflow interruptions increase simultaneously with autonomy, especially with regard to digital communication devices. Role expectations and opportunities for development depend on how the profession and the technology relate to each other, especially when working with automated systems. The implications for the work demands necessary to deal with changes in work characteristics include knowledge about technology, openness toward change and technology, skills for self- and time management and for further professional and career development. Implications for the design of formal learning environments (i.e., the content, method, assessment, and guidance) include that the work demands mentioned must be part of the content of the trainings, the teachers/trainers must be equipped to promote those work demands, and that instruction models used for the learning environments must be flexible in their application.

## Introduction

In the face of technology-driven disruptive changes in societal and organizational practices, continuous vocational education and training (CVET) lacks information on how the impact of technologies on work must be considered from an educational perspective (Cascio and Montealegre, 2016). Research on workplace technologies, i.e., tools or systems that have the potential to replace or supplement work tasks, typically are concerned with one out of two areas of interest: First, economic and sociological research repeatedly raises the question on technological mass-unemployment and societal inequality as a result of technological advances (Brynjolfsson and McAfee, 2014; Ford, 2015; Frey and Osborne, 2017). And second, management literature questions the suitability of prevailing organizational structures in the face of the so-called “fourth industrial revolution” (Schwab, 2017), taking visionary leaps into a fully automated future of digital value creation (Roblek et al., 2016).

Many of the contributions of scholars discuss the enormous potential of new technologies for work and society at a hypothetical level, which led to a large number of position papers. Moreover, the question on what consequences recent developments, such as working with robots, automated systems or artificial intelligence will have for different professions remain largely unclear. By examining what workplace technologies actually “do” in the work environment, it was suggested that *work tasks* change because of technological developments (Autor et al., 2003; Autor, 2015). This is due to technologies substituting different operations or entire tasks and thus leave room for other activities. Jobs are defined by the work tasks and the conditions under which the tasks have to be performed. This in turn defines the necessary competences, that is the potential capacity to carry out a job (e.g., Ellström, 1997). Therefore, CVET needs to be informed on the changes that technology causes in work tasks and the consequential characteristics of work. Only then CVET is able to derive the required competences of employees and organize learning environments that foster the acquirement of these competences. These insights can be used to determine the implications thereof for the components of formal learning environments: content, didactics, trainer behavior, assessment, and resources (e.g., Mulder et al., 2015).

The aim of this systematic literature review is to get insight into the effects of new technological developments on work characteristics in order to derive the necessary work demands and their implications for the design of formal learning environments in CVET.

Therefore, the following research questions will be answered:

RQ_1_: What are the effects of new technologies on work characteristics?
RQ_2_: What are the implications thereof for continuous vocational education and training?

Theoretical considerations on the relationships between technology and work characteristics are presented before the methods for searching, selecting and analyzing suitable studies are described. Regarding the results section, the structure is based on the three main steps of analyzing the included studies: First, the variables identified within the selected studies are clustered and defined in terms of work characteristics. Second, a comprehensive overview of evidence on the relationships between technologies and work characteristics is displayed. Third, the evidence is evaluated regarding the work demands that result from technologies changing work characteristics. Finally, the implications for CVET and future research as well as the limitations of this study will be discussed.

## Theoretical Framework

In this section, a conceptualization of technology and theoretical assumptions on relationships between technology and work characteristics will be outlined. Research within various disciplines, such as sociology, management, economics, educational science, and psychology was considered to inform us on the role of technology within work. Completing this section, an overview of the various components of learning environments is provided to be used as a basis for the analyses of the empirical evidence.

### Outlining Technology and Recent Technological Developments

A clear definition of technology often lacks in studies, what may be due to the fact that the word itself is an “equivoque” (Weick, 1990, p. 1) and a “repository of overlapping inconsistent meanings” (McOmber, 1999, p. 149). A suitable definition can be provided by analyzing what technologies actually “do” (Autor et al., 2003, p. 1,280). The primary goal of technology at work is to save or enhance labor in the form of work tasks, defined as “a unit of work activity that produces output” (Autor, 2013, p. 186). Technology can therefore be defined as mechanical or digital devices, tools or systems. These are used to replace work tasks or complement the execution of work tasks (e.g., McOmber, 1999; Autor et al., 2003). According to this view, technology is conceptualized according to “its status as a tool” (“instrumentality”; McOmber, 1999, p. 141). Alternatively, technology is understood as “the product of a specific historical time and place,” reflecting a stage of development within a predefined historical process (“industrialization”; McOmber, 1999, p. 143) or as the “newest or latest instrumental products of human imagination” (“novelty”; McOmber, 1999, p. 143), reflecting its nature that is rapidly replacing and “outdating” its predecessors. The definition according to “instrumentality” is particularly suitable for this research, as the interest focuses on individual-level effects of technologies and its use for accomplishing work. Therefore, the technology needs to be mentioned explicitly (e.g., “robot” instead of “digital transformation”) and described specifically in the form with which the employee is confronted at the workplace. Different definitions may reflect different perspectives on the role of technology for society and work. These perspectives in the form of paradigmatic views (Liker et al., 1999) include philosophical and cultural beliefs as well as ideas on organizational design and labor relations. They differ with regard to the complexity in which the social context is believed to determine the impact of technology on society. Listed in accordance to increasing social complexity, the impact may be determined by technology itself (i.e., “technological determinism”), established power relations (i.e., “political interest”), managerial decisions (i.e., “management of technology”), or the interaction between technology and its social context (i.e., “interpretivist”) (Liker et al., 1999). Later research added an even more complex perspective, according to which the effects of technology on society and organizations are determined by the relations between the actors themselves (i.e., “sociomateriality”; Orlikowski and Scott, 2008). Paradigmatic views may guide research in terms of content, purpose and goals, which in turn is likely to affect the methods and approach to research and may be specific to disciplines. For instance, Marxist sociological research following the view of “political interest” or research in information systems following the view of “management of technology.”

New technological developments are widely discussed in various disciplines. For instance, Ghobakhloo (2018) summarizes the expected areas of application of various technological concepts within the “smart factory” in the manufacturing industry: The internet of things as an umbrella term for independent communication of physical objects, big data as procedure to analyse enormous amounts of data to predict the consequences of operative, administrative, and strategic actions, blockchain as the basis for independent, transparent, secure, and trustworthy transaction executed by humans or machines, and cloud computing as an internet-based flexible infrastructure to manage all these processes simultaneously (Cascio and Montealegre, 2016; Ghobakhloo, 2018). The central question to guide the next section is to what extent these new technologies, and also well-established technologies such as information and communication technologies (ICT), which are constantly being expanded with new functions, could influence work characteristics on a theoretical basis.

### Theories on the Relationships Between Technology and Work Characteristics

A central discussion on technology can be found in the sociological literature on deskilling vs. upgrading (Heisig, 2009). The definition of “skill” in empirical studies on this subject varies regarding its content by describing either the level of complexity that an employee is faced with at work, or the level of autonomy that employees are able to make use of Spenner (1990). Theories advocating the deskilling of work (e.g., labor process theory; Braverman, 1998) propose that technology is used to undermine workers' skill, sense of control, and freedom. Employees need to support a mechanized workflow under constant surveillance in order to maximize production efficiency (Braverman, 1998). Other authors, advocating “upskilling” (Blauner, 1967; Bell, 1976; Zuboff, 1988), propose the opposite by claiming that technology frees employee's from strenuous tasks, leaving them with more challenging and fulfilling tasks (Francis, 1986). In addition, issues of identity at work were raised by Blauner (1967) who acknowledged that employees may feel “alienated” as soon as technologies change or substitute work that is meaningful to them, leaving them with a feeling of powerlessness, meaninglessness, or self-estrangement (Shepard, 1977). In sum, sociological theories suggest that technology has an impact on the level of freedom, power and privacy of employees, determining their identity at work and the level of alienation they experience.

According to contingency theories (Burns and Stalker, 1994; Liker et al., 1999) technology is a means to reduce uncertainty and increase competitiveness for organizations (Parker et al., 2017). Therefore, the effects of technology on the employee depend on strategic decisions that fit the organizational environment best. When operational uncertainty is high, organizations get more competitive by using technology to enhance the flexibility of employees in order to enable a self-organized adaption to the changing environment (Cherns, 1976). This increases employee's flexibility by allowing them to identify and decide on new ways to add value to the organization (“organic organization”; Burns and Stalker, 1994). When operational uncertainty is low, organizations formalize and standardize procedures in order to optimize the workflow and make outputs more calculable (“mechanistic organization”; Burns and Stalker, 1994). This leads to less opportunities for individual decision-making and less flexibility for the employees. In sum, contingency theories suggest, that the effects of technology depend on the uncertainty and competitiveness in the external environment and may increase or decrease employee's flexibility and opportunities for decision-making and self-organization.

Economic research following the task-based approach from Autor et al. (2003) suggests, that technology substitutes routine tasks and complements complex (or “non-routine”) ones. Routine manual and cognitive tasks usually follow a defined set of explicit rules, which makes them susceptible to automation. By analyzing qualification requirements in relation to employment rates and wage development, it was argued that workplace automation substitutes routine and low-skill tasks and thus favors individuals who can carry out high-skilled complex work due to their education and cognitive abilities (Card and DiNardo, 2002; Autor et al., 2003). This means, that the accomplishment of tasks “demanding flexibility, creativity, generalized problem-solving, and complex communications” (Autor et al., 2003, p. 1,284) becomes more important. Complex tasks, so far, posed a challenge for automation, because they required procedural and often implicit knowledge (Polanyi, 1966; Autor, 2015). However, recent technological developments such as machine learning, are capable of delivering heuristic responses to complex cognitive tasks by applying inductive thinking or big data analysis (Autor, 2015). Regarding complex manual tasks, mobile robots are increasingly equipped with advanced sensors which enable them to navigate through dynamic environments and interactively collaborate with human employees (Cascio and Montealegre, 2016). In sum, economic research following the task-based approach argues that technology affects the routineness and complexity of work by substituting routine tasks. However, new technologies may be able to increasingly substitute and complement not only routine tasks, but complex tasks as well. According to the theories, this will again increase the complexity of work by creating new demands for problem-solving and reviewing the technology's activity.

Useful insights can be gained from psychological theories that explicitly take the role of work characteristics into account. Work characteristics are often mentioned by for instance sociological theories (e.g., autonomy and meaningfulness) without clearly defining the concepts. Particularly the job characteristics model of Hackman and Oldham (1975) and the job-demand-control model of Karasek (1979) and Karasek et al. (1998) are consulted to further clarify the meaning of autonomy and meaningfulness at work. With regard to autonomy, Hackman and Oldham's model 1975 conceptualizes autonomy as a work characteristic, defined as “the degree to which the job provides substantial freedom, independence, and discretion to the employee in scheduling the work and in determining the procedures to be used in carrying it out” (Hackman and Oldham, 1975, p. 162). According to the authors, autonomy facilitates various work outcomes, such as motivation and performance. In a similar vein, Karasek et al. (1998) stress the role of autonomy in the form of “decision authority” that interacts with more demanding work characteristics, such as workload or frequent interruptions and therefore enables a prediction of job strain and stress (Karasek et al., 1998). With regard to meaningfulness, Hackman and Oldham (1975) clarify that different core job dimensions, such as the significance of one's own work results for the work and lives of other people, the direct contribution to a common goal with visible outcomes, and the employment of various skills, talents and activities all enhance the perception of meaningfulness at work. In sum, psychological theories on employee motivation and stress clarify the concepts of autonomy and meaningfulness by illustrating the factors that contribute to their experience in relation to challenging and rewarding aspects of work.

### Components of CVET

In order to formulate the implications for CVET of the studied effects of technology on work characteristics, a framework with the different components of CVET is needed. The objective of the VET system and continuous education is to qualify people by supporting the acquirement of required competences, for instance by providing training. Competences refer to the potential capacity of an individual in order to successfully carry out work tasks (Ellström, 1997). They contain various components such as work-related knowledge and social skills (e.g., Sonntag, 1992). Competences are considered here as “the combination of knowledge, skills and attitude, in relation to one another and in relation to (future) jobs” (Mulder and Baumann, 2005, p. 106; e.g., Baartman and de Bruijn, 2011).

**Figure 1:**
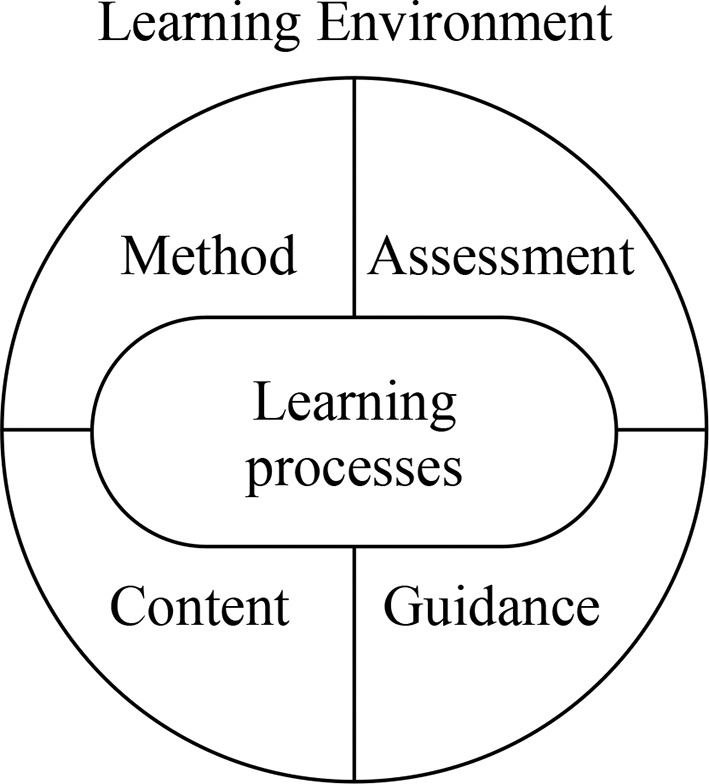
Components of CVET learning environments (adapted from Mulder et al., 2015, p. 501).

Participants in CVET enter the system with competences, such as prior knowledge, motivation, and expectations. It is argued that these have to be considered when designing learning environments for CVET. Next to making the distinction between the different components of learning environments content, guidance, method, and assessment, it is considered important that these components are coherent and consistent (Mulder et al., 2015). For instance, the content of the training needs to fit to the objectives and the background of the participants. The same goes for the method or didactics used (e.g., co-operative learning, frontal instruction) and the guidance of teachers, mentors or trainers. In addition, assessment needs to be consistent with all these components. For instance, problem based learning or competence based training requires other forms of assessment than more classical teacher centered forms of didactics, which makes a classic multiple choice test not fitting (Gulikers et al., 2004). Figure 1 contains an overview of the components of learning environments for CVET.

## Methods

Three steps are necessary to answer the research questions. Firstly, a systematic search and review of empirical studies reporting evidence on the direct relationships between new technologies and work characteristics. Secondly, an analysis of the evidence with regard to its implications for work demands. Thirdly, deriving the work demands and their implications for CVET.

### Systematic Search Strategy

Due to the interdisciplinary nature of our research, specific databases were selected for each of the disciplines involved: Business Source Premier (business and management research) and PsycArticles (psychology) were searched via EBSCOhost, and ERIC (educational science), and Sociological Abstracts (sociology) were searched via ProQuest.

Identifying suitable keywords for technological concepts is challenging due to the rapidly changing and inconsistent terminology and the nested nature of technological concepts (Huang et al., 2015). Therefore, technological terms were systematically mapped by using the different thesauri provided by each of the chosen databases. After exploding a basic term within a thesaurus, the resulting narrower terms and related terms were documented and examined within the following procedure: (a) Checking the compatibility with our definition of technology reflecting its instrumentality, (b) Adjustment of keywords that are too broad or too narrow, (c) Disassembling nested concepts. The procedure was repeated stepwise for each of the databases. Finally, 45 terms that reflect new technologies were documented and used for the database search.

Keywords reflecting work characteristics are derived from the theoretical conceptualizations previously outlined. Synonyms for different concepts within the relevant theories were identified and included. In order to narrow our search results, additionally operators for empirical studies conducted in a workplace setting were added.

In order to avoid unnecessary redundancy, the use of asterisks was carefully considered, provided that the search results did not lose significantly in precision or the number of hits did not grow to an unmanageable number of studies. The final search string is shown in Table 1.

**Table 1:** Final search string.

	**Search terms**
Search terms for technology (Subject terms)	“artificial intelligence” or robot^*^ or “big data” or computer^*^ or “mobile device” or “wearable technology” or “implant” or “cloud computing” or “virtual reality” or “augmented reality” or blockchain or “automated manufacturing” or wireless or “data processing” or “real-time” or smart or cyber^*^ or “assistive technology” or “instant messaging” or “social media” or “mobile communication” or ICT^*^ or “information and communication technology” or “machine learning” or avatar^*^ or “RFID” or “digital device^*^” or “mobile device^*^” or virtual^*^ or “autonomous driving” or digitization or digitalization or digitisation or digitalisation or “information technology” or internet or smartphone or sensor^*^ or “cyber-physical-system” or “internet of things” or IoT or “mobile internet” or “cloud technology” or “automated system” or “workplace automation” AND
Search terms for work characteristics	Meaning^*^ or meaningfulness or complexity or flexibility or routine^*^ or “non-routine^*^” or “job demand” or intensity or workload^*^ or workflow or pressure or privacy “skill variety” or “task variety” or “skill diversity” or “task diversity” or responsibility or autonom^*^ or control^*^ or “decision-making” or freedom or alienation or identity or power or competition or uncertainty or “job characteristics” or “work characteristics” or “task characteristics” or “work environment” AND
Restriction: Empirical evidence (Abstract)	empirical^*^ or quantitative or qualitative or survey^*^ or “case study” or questionnaire^*^ or interview^*^ or evidence AND
Restriction: Work context	Workplace or job or career or employment

### Eligibility Criteria and Study Selection

Technical criteria included methodological adequacy. This was ensured by only including studies published in peer-reviewed journals. In addition, the studies had to provide quantitative or qualitative data on relationships between technology and work characteristics. Only English-language studies were considered, because most of the studies are published in English and therefore the most complete overview of the existing knowledge on this topic can be obtained. This also enables as many readers as possible to have access to the original studies and analyse the findings of the empirical studies themselves.

Concerning technology, variables had to express the direct consequence or interaction with a certain technology (e.g., the amount of computer-use or experience with robots in the workplace) and indirect psychological states that conceptually resulted from the presence of the technology (e.g., a feeling of increased expectations concerning availability). Regarding work characteristics, variables had to describe work-related aspects associated with our conceptualization of work characteristics (e.g., a change in flexibility or the perception of complexity).

Regarding the direction of effects, only studies that focused on the implementation or use of technologies for work-related purposes were included. Studies were excluded, if they (a) tested particular designs or features of technologies and evaluated them without considering effects on work characteristics, (b) regarded technology not as a specific tool but an abstract process (e.g., “digital transformation”), (c) were published before 1990 due to the fact that the extent of usability and usefulness of technologies before that time should be substantially limited compared to today (e.g., Gattiker et al., 1988), and (d) investigated the impact of technologies on society in general without a specific relation to professional contexts (e.g., McClure, 2018).

Studies that were found but that did not report empirical findings on the relationships between technology and work characteristics, but rather on the relationships between technology and work demands (e.g., specific knowledge or skills) or work outcomes (e.g., performance, job satisfaction) were documented. Since the aim for this study was to derive the work demands from the work characteristics in any case, the studies that reported a direct empirical relationship between technology and work demands were analyzed separately (*N* = 7).

### Data Extraction

The variables expressing technology and work characteristics were listed in a table, including the quantitative or qualitative data on the relationships. Pearson's r correlations were preferred over regression results to ensure comparability. For qualitative data, the relevant passages documenting data were included. Finally, methodological information as well as sample characteristics and size are listed.

### Analysis of the Results

Firstly, the variables containing work-related aspects are clustered thematically into a comprehensive final set of work characteristics. This is necessary to reduce complexity due to variations in naming, operationalization and measurement and to make any patterns in the data more visible. Deviations from the theoretically expected clusters are noted and discussed before synthesizing the evidence narratively in accordance to the research questions (Rodgers et al., 2009). As proposed, the evidence on changing work characteristics is analyzed with respect to the resulting work demands in the sense of knowledge, skills, attitude and behavior, which in turn are used to determine the implications for the different components of CVET.

## Results

**Figure 2:**
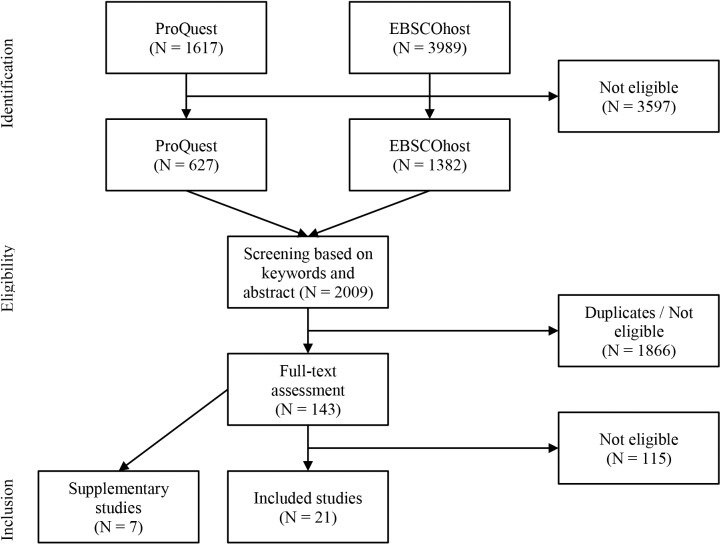
Flowchart of literature search process.

Figure 2 depicts a flowchart documenting the literature search. In sum, 21 studies providing evidence on relationships between technology and work characteristics were included. In addition, seven supplementary studies containing empirical evidence on relationships between technology and specific work demands were identified. These studies are taken into account when deriving the work requirements. Next, the descriptive characteristics of the included studies will be reported. After that, the evidence on relationships between technologies and work characteristics of the 21 included studies will be summarized, before finally deriving the work demands based on the evidence found.

### Characteristics of Studies

Table 2 contains an overview of the characteristics of selected studies. Most of the studies were published between 2015 and 2019 (52%). Nearly half of the studies were conducted in Europe (48%), followed by North America (33%). Most of the studies reported qualitative data collected with methods such as interviews (62%).

**Table 2:** Characteristics of the studies.

**Characteristic**	**Number of studies**
Publication year	
2015–2019	11
2010–2014	7
2005–2009	1
2000–2004	1
1990–1999	1
Domain	
Health care/Pharmaceutical industry/Therapy	5
Telecommunication/Technology	2
Manufacturing/Automobile/Port terminal	4
Accounting/Government/Postal service	3
Various	7
Origin	
North America	7
Asia	1
Europe	10
Australia	2
Africa	1
Design	
Survey study	6
Secondary analysis	2
Interview study	5
Case study	3
Action research	1
Other qualitative approach	4
Instruments	
Questionnaires	9
Interviews	10
Focus groups	3
Observations	2
Internal data (documents, log-data)	2
Workshop discussion	1
Data	
Quantitative	8
Qualitative	13

*N = 21*.

The studies investigated a variety of technologies, such as computers (1, 7), various forms of Information and Communication technologies (ICTs; 2, 3, 17, 18, 21) in a broad sense, including specific examples of work-extending technologies and other tools for digital communication, information technology (IT) systems supporting information dissemination and retrieval within organizations (4, 9), automated systems supporting predominantly physical work procedures (5, 6, 11, 12, 13, 14, 20), robots (15, 19), social media enabling professional networking and participation in organizational and societal practices (8, 16), and more domain-specific technologies such as clinical technology supporting professional decisions (9) and field technology for labor management (10).

### Relationships Between Technology and Work Characteristics

In sum, nine work characteristics were identified and defined distinctively. Table 3 contains the operational definitions of the final work characteristics and the work-related aspects they consist of. The final work characteristics are: Workflow interruptions, workload, manual work, mental work, privacy, autonomy, complexity, role expectations, and opportunities for development.

**Table 3:** Overview for final work characteristics and the exemplary work-related aspects assigned to them.

**Work characteristic**	**Operational definition**	**Examples of measured work-related aspects affected by technology**
Workflow interruptions	Extent to which employees can focus on a single task and avoid interruptions	Level of interruptions
		Quality of workflow
		Quality of communication processes
		Level of multitasking
		Need for multitasking
Workload	Amount and pace of work	Work overload
		Job demands (workload pressure)
		Work pressure
		Level of job speed
		Time pressure
Manual work	Extent to which the environment is characterized by physical tasks and requirements	Facilitation of physical tasks
		Content and scope of routine work tasks
		Amount of physically demanding tasks
		Physical demands
Mental work	Extent to which the environment is characterized by mental/cognitive tasks and requirements	Diagnosing and navigating demand
		Amount of monitoring tasks
		Problem-solving demand
		New challenging mental tasks
Privacy	Extent to which employees have control over their public image and their personal data at work	Invasion of privacy
		Perceived managerial surveillance
		Managerial tracking of behavior
		Control over work-related data
		Peer-monitoring
Autonomy	Extent to which there is discretion regarding the type, order, methods, or time in which work needs to be done	Method-order autonomy
		Job decision latitude
		Time-method control
		Flexibility
		Instant accessibility of people and knowledge
		Job enrichment
Role expectations	Extent to which the job matches one's own and others' expectations regarding the role and the meaning associated with it	Role ambiguity
		Role expansion
		Role specific inner obligation for availability
		Connectivity or networking pressure
		Production responsibility
		Meaningful content of work
Complexity	Extent to which there is a lack of structure and transparency resulting from highly diverse and interconnected tasks and the associated ambiguity	Complexity
		Job complexity
		Situational awareness
Opportunities for development	Extent to which work provides opportunities for self-development and the need for development of skill and knowledge	Opportunities for skill and knowledge-acquisition
		Opportunities for professional development
		Continuous qualification demands
		Requirement to stay up to date with new technologies

The complete overview of the selected studies and results for the relationships between technology and work characteristics is provided in Table 4 (for quantitative data) and Table 5 (for qualitative data). To further increase comprehensibility, the variables within the tables were labeled according to their function in the respective study (e.g., independent variable, mediating variable, dependent variable; see notes).

**Table 4:** Studies providing quantitative evidence for the relationship between technology and work-related aspects.

		**Relationship between technological and work-related aspects**				**Methodology**		
	**Technology under study**	**Expression of technology**	**Work-related aspects**	**Correlation/Effect**	**Work characteristics**	**Study design**	***N* (Domain)**	**References**
1	Computer	Amount of computer work (IV)	Workload (IV)	*r* = 0.06^**^	Workload	2, b, i	*N* = 18,723 (various domains; Europe)	Kraan et al., 2014
			Method-order autonomy (IV)	*r* = 0.21^**^	Autonomy			
		Technological pacing (IV)	Workload	*r* = 0.20^**^	Workload			
			Method-order autonomy	*r* = −0.19^**^	Autonomy			
2	ICT	Presenteeism (IV)	Invasion of privacy (ME)	*r* = 0.13^**^	Privacy	1, a, i	*N* = 661 (Various domains; United States)	Ayyagari et al., 2011
			Work overload (ME)	*r* = 0.19^**^	Workload			
			Role ambiguity (ME)	*r* = 0.14^**^	Role expectations			
		Anonymity (IV)	Invasion of privacy	*r* = −0.32^**^	Privacy			
			Work overload	*r* = −0.14^**^	Workload			
			Role ambiguity	*r* = −0.08	Role expectations			
		Pace of change (IV)	Invasion of privacy	*r* = 0.16^**^	Privacy			
			Work overload	*r* = 0.20^**^	Workload			
			Role ambiguity	*r* = 0.25^**^	Role expectations			
3	ICT	Email usage beyond the workplace (IV)	Job decision latitude (DV)	*B* = 0.02	Autonomy	1, a, i	*N* = 703 (various domains; United States)	Chen and McDonald, 2015
		Phone usage beyond the workplace (IV)	Job decision latitude	*B* = 0.13^*^	Autonomy			
		Positive ICT impacts (Productivity, flexibility, collaboration, connection) (IV)	Job decision latitude	*B* = 0.97^*^	Autonomy			
		Negative ICT impacts (long hours, job stress, stickiness, distraction) (IV)	Job decision latitude	*B* = −0.14^*^	Autonomy			
4	Information technology system (ERP)	Frequency of computer usage (IT use) (IV)	Complexity after ERP implementation (DV)	*r* = 0.37^*^	Complexity	1, a, ii	*N* = 44 (Accounting)	Marler and Liang, 2012
		ERP use (IV)	Complexity after ERP implementation	*r* = 0.33^*^	Complexity			
5	Letter sorting machine	Machine-paced work (IV)	Autonomy (ME)	*r* = −0.46^**^	Autonomy	1, a, i	*N* = 4682 (postal service industry; United States)	Amick and Celentano, 1991
			Job demands (Workload pressure) (ME)	*r* = 0.32^**^	Workload			
6	Automated Manufacturing Technology	Technological coupling (Complexity of machine) (IV)	Time method control (IV)	*r* = −0.23^*^	Autonomy	1, a, i	*N* = 216 (Manufacturing; Israel)	Dvash and Mannheim, 2001
			Monitoring demand (IV)	*r* = 0.01	Mental work			
			Problem-solving demand (IV)	*r* = 0.03	Mental work			
			Production responsibility (IV)	*r* = 0.11	Role expectations			
			Job enrichment (IV)	*r* = 0.08	Autonomy			
7	Computer	Daily computer-use for work (IV)	Level of job speed (ME)	*B* = 0.14^*^	Workload	2, a, i	*N* = 2556 (various domains; United States)	Chesley, 2014
			Level of job interruptions (ME)	*B* = 0.48^**^	Workflow interruptions			
			Level of multitasking (ME)	*B* = 0.31^**^	Workflow interruptions			
			Job autonomy (CV)	*r* = 0.07†	Autonomy			
			Job complexity (CV)	*r* = 0.14†	Complexity			
8	Social media	Frequency of social networking service use for work-related communication (IV)	Work pressure (ME)	*r* = 0.32^*^	Workload	1, a, i	*N* = 364 (telecommunication and consulting; Netherlands)	van Zoonen and Rice, 2017
			Autonomy (ME)	*r* = 0.14^*^	Autonomy			

*IV, independent variable; ME, mediating variable; DV, dependent variable; CV, control variable; r, Pearson's r correlation; B, unstandardized coefficients; Concerning methodology: 1, survey; 2, secondary analysis; a, questionnaires; b, interviews; i, cross-sectional; ii, longitudinal/multiple measurements*;

* *p < 0.05*;

** *p < 0.01*;

† *No level of significance reported*.

**Table 5:** Studies providing qualitative evidence for the relationship between technology and work-related aspects.

	**Technology**	**Research objective**	**Work-related aspects affected by technology**	**Work characteristics**	**Study design**	***N* (domain)**	**References**
9	Clinical technology (CT)	Exploration of nurses' perceptions of new technology in relation to their skills, autonomy and experience of work	Increased workload due to higher efficiency and patient flow rates accomplished by CT use	Workload	1, a, i	*N* = 125 (Nursing; Australia)	Gough et al., 2014
			Increased complexity of interventions due to enhanced treatment potential	Complexity			
			Enhanced opportunities for clinical knowledge- and skill-acquisition due to clinical information provided by CT in a high dependency environment	Opportunities for development			
	Information technology (IT)		Reduced content of work (e.g., time with patient care) due to higher reporting requirements by IT-system (resulting in time spent with technology)	Role expectations			
			No feeling of managerial surveillance or control by IT database	Privacy			
10	Field technology	Exploration if employees' right for data privacy is challenged in the face of electronic governance and control by management	Increased control by management through continuous tracking of behavior and performance and managerial allocation of work (labor management)	Privacy	1, a, e, i	*N* = 90 (Installation, cleaning, home care, security, transport; Norway)	Tranvik and Bråten, 2017
			Less control over work-related data by employees due to automatic and non-transparent data transmission	Privacy			
11	Automated terminals	Understanding how work, job roles and associated skills have changed across technological shifts	Facilitation and acceleration of routine and physical work tasks through process automation controlled by computer technologies	Manual work	4, a, d, ii	Unreported (Port terminal work; Australia)	Gekara and Thanh Nguyen, 2018
			Higher diagnosing and navigating tasks within a digitized work environment including mobile and fixed digital devices	Mental work			
			Decreased content and scope of operational tasks and increase in monitoring tasks due to digital devices	Manual work			
12	Automated production systems	Exploration of the relationship between the quality of human-machine interaction and work satisfaction, workload and stress	Increased time pressure and need for multitasking due to technical interruptions	Workflow interruptions	1, a, i	*N* = 36 (Manufacturing industry; Germany)	Körner et al., 2019
			Occasional low situational awareness due to increased system complexity and inadequate information	Complexity			
			Continuous qualification requirements to deal with complex computer-related problems and expectation of learning-by-doing	Opportunities for development			
13	Bar-coded medication administration technology	Investigating the impact of automated medication administration technology on nurses' problem-solving behavior	Increased workload due to the technology blocking familiar problem-solving strategies	Workload	4, a, d, i	*N* = 17 for observations; *N* = 45 for interviews (Nursing; United States)	Holden et al., 2013
			Increasing occurrence of new problems that require creative problem-solving and “workarounds” by using or bypassing the system	Mental work			
14	Automated dispensing system	Determining the effects of installing an automated dispensing system on staff experience of work, psychological contract, individual outcomes and future plans	Opportunities for role expansion due to the opportunity to approach new value-adding tasks through automation	Role expectations	2, c, ii	*N* = 31 (Pharmaceutical industry; United Kingdom)	James et al., 2013
			Increasing physical demands for employees directly working with the system due to monotony and time pressures	Manual work			
			Reduction of roles for employees that support the system directly (e.g., technicians)	Role expectations			
15	Cobots	Identifying issues for the African workforce regarding the implementation of cobots	Decreased amount of physically demanding tasks due to physical support by cobots	Manual work	4, b, i	*N* = 12 (Automobile manufacturing; Africa)	Calitz et al., 2017
			Increase in new and challenging mental tasks that require a certain understanding, acceptance and trust toward cobots	Mental work			
16	Social media	Explication of the boundary-related rules regarding personal and professional social media use at work	Perceived feeling of peer-monitoring and judgement in case of personal social media use (e.g., Facebook, Twitter)	Privacy	2, a, i	*N* = 29 (Technology sector; United States)	Walden, 2016
			Perceived pressure to network with peers and clients with professional social media due to acceptance and positive appraisal of work-related use (e.g., LinkedIn)	Role expectations			
17	Tools for digital communication	Determining the relationship between communication in the digital work environment and wellbeing at work, factors influencing the relationship, and its context	Interruption of workflow and attention due to the constant flow of messages and communication via various communication platforms and devices	Workflow interruptions	3, e, f, i	*N* = 36 (Industrial, insurance, finance)	Bordi et al., 2018
			Requirement to stay up to date with new tools for digital communication due to changing technology and customer preferences	Opportunities for development			
			Increased flexibility (time, place, task) due to self-initiated multitasking and use of various tools	Autonomy			
18	Work-extending technologies	Examining the effects of work-extending technologies on working time, relationships, and strategies employed by employees to deal with technological effects and its impact	Increased efficiency, productivity and flexibility (working from home or while traveling) if work extending technologies can be used	Autonomy	1, a, c, i	*N* = 61 (Government department; Canada)	Towers et al., 2006
			Higher workload due to perceived expectations for constant availability and longer work days	Workload			
19	Robots	Exploring therapists' needs regarding robots and functions that make robots perceived as useful	Potential workflow support if robots support repeatable tasks, observe behavior and give objective feedback	Workflow interruptions	4, b, i	*N =* 21 (Autism therapy; Poland)	Zubrycki and Granosik, 2016
			No creation of opportunities to improve therapist value or for professional self-development by robots	Opportunities for development			
20	Robot-facilitated pharmacy distribution	Understanding to what extent employers considered job quality in advance to an automated system's introduction and how those considerations impacted various aspects of job quality for employees	More opportunities for upskilling and interdisciplinary learning through job rotation as a consequence of the system's introduction (in general)	Opportunities for development	2, a, c, i	*N* = 45 (Health care; United Kingdom)	Findlay et al., 2017
			Increase in meaningful job content (less repetitive work, greater task variety, more interaction with colleagues and patients) for ward-based employees whose work is strongly supported by the automated system (i.e., ward-based technicians and support staff)	Role expectations			
			Higher paced, more repetitive and less skilled work for employees that support the system directly (i.e., dispensary technicians)	Role expectations			
			Less possibilities for learning and career development due to decreased opportunities for job rotation for some employees (i.e., dispensary technicians)	Opportunities for development			
21	ICT	Examination of specific stressors and benefits resulting from work-related technology-use in public relations, journalism and advertising	Regular interruptions of workflow due to constant availability via mobile communication	Workflow interruptions	1, a, i	*N* = 25 (Advertising, public relations, journalism; Austria, Hong Kong)	Ninaus et al., 2015
			Connectivity pressure as a consequence of increased response expectations due to the mobile wireless communication	Role expectations			
			Inner obligation for availability as a result of being able to perform and compete at any time	Role expectations			
			Improved workflow and improved communication as a result of instant communication detached from workplace and working time	Workflow interruptions			
			Instant accessibility of people and knowledge as a result of flexible device-use and integration of various digital platforms and devices	Autonomy			
			Increased flexibility as a result of mobile technologies allowing to autonomously allocate working time and place	Autonomy			

*Concerning methodology: 1, interview study; 2, case study; 3, action research; 4, other qualitative approaches; a, interviews; b, questionnaire; c, focus groups; d, observations; e, internal data (documents, log-data); f, workshop-discussion; i, cross-sectional; ii, longitudinal*.

#### Complexity

There is quantitative evidence on positive relationships between IT system use and complexity reported by two studies (4, 9). On a similar note, qualitative evidence suggests lower situational awareness within automated systems indicating an increase in complexity (12), and clinical technology being associated with an increase in complexity for nurses (9).

#### Autonomy

There is mixed quantitative evidence on the relationships between computer work and autonomy (1). The amount of computer work is positively related to autonomy, while technological pacing is negatively related to autonomy. Working within automated systems is negatively (5, 6) or not related (6) to different measures of autonomy. ICT use shows mixed relationships with job decision latitude (3) depending on ICT features that describe negative or positive effects of use. Evidence indicates a positive relationship between social media use and autonomy. Qualitative evidence suggests that ICT use increases autonomy (21) and flexibility (17, 18, 21).

#### Workload

Quantitative studies indicate strong positive relationships between computer work (1) and ICT use (2) and workload. The relationships are not consistent due to the fact that certain ICT features differ in their effects on workload. ICT characteristics such as presenteeism and pace of change are positively related to feelings of increasing workload, while a feeling of anonymity is negatively associated with workload. Evidence indicates positive relationships between time or workload pressure in the context of computer work (7), working in an automated system (5), as well as social media use (8) and provide evidence for positive relationships between various technologies and workload. Qualitative studies report similar outcomes. ICT use (18), automated systems (12, 13) as well as clinical technology (9) are reported to increase the workload.

#### Workflow Interruptions

Quantitative evidence indicates positive relationships between computer work and increasing levels of interruptions as well as an increasing demand for multitasking (7). Qualitative evidence suggests that ICT use is positively associated with an increased level of interruptions on the one hand and workflow support on the other hand (21). Further qualitative evidence suggests that robots at the workplace have positive effects on workflow support (19), and automated systems seem to increase the level of multitasking required in general (12).

#### Manual Work

Qualitative evidence suggests a decrease in the amount of physically demanding tasks when working with automated systems (11) and robots (15). In one study, qualitative evidence suggests an increase in manual work for technical jobs where automated systems are used (14).

#### Mental Work

Quantitative evidence indicates no relationships between monitoring tasks or problem-solving demands for technical jobs within automated systems (6). Qualitative evidence however suggests positive relationships between work within automated systems and various cognitive tasks and demands, such as problem-solving and monitoring (11, 13), while working with robots increases the amount of new and challenging mental tasks (15).

#### Privacy

Quantitative evidence indicates that different ICT characteristics show different relationships with invasion of privacy (2). Some features are negatively related to invasion of privacy (anonymity) and others are positively related to it (presenteeism, pace of change). Qualitative evidence suggests that IT systems are not related to the perception of managerial surveillance (9), while social media is positively related to peer-monitoring (16), and field technology is negatively related to employee data control (10).

#### Role Expectations

Quantitative evidence indicates that ICT use is inconsistently related to role ambiguity depending on specific characteristics of the technology (2). Regarding automated systems, quantitative evidence indicates no relationship between working in an automated system and opportunities for role expansion in the form of an increased perceived responsibility (6). Qualitative evidence suggests that ICT use increases the expectations for availability and connectivity (21), and social media positively affects networking pressure (16). Qualitative evidence suggests that IT systems (9) decrease meaningful job content and role expansion. Qualitative evidence suggests that automated systems vary with regard to enhancing meaningfulness at work, dependent on whether the work tasks are complemented by the system or revolve around maintaining the system (20).

#### Opportunities for Development

Qualitative evidence suggests that ICT use (12) as well as working with an automated system (17) increase the demands for continuing qualification. Qualitative evidence suggests that opportunities for learning and development are prevalent with clinical technology (9) and absent when working with robots (19). Mixed qualitative evidence regarding automated systems and learning opportunities suggests that the effects depend on the differences in work roles in relation to being supported by the system or supporting the system (20).

A comprehensive summary of the outcomes can be found in Table 6. The information in this table gives a summary of the evidence found for the different technologies and their relationships to work characteristics, more specifically to work related aspects. Important distinctive characteristics such as sample characteristics are listed in Tables 4, 5.

**Table 6:** Overview over identified relationships between technology and work characteristics.

**Number of study**	**Work-related aspects affected by technology (clustered)**	**Work characteristic**	**Computer**	**ICTs**	**IT systems**	**Automated systems**	**Social media**	**Clinical technology**	**Field technology**	**Robots**
7, 17	Level of interruptions	Workflow interruptions	⊕	+						
19, 21	Support of workflow			−+						+
7, 12	Level of multitasking		⊕			+				
2, 1, 9, 13, 18	Workload	Workload	⊕⊕	⊕⊖⊕+		+		+		
5, 8	Workload pressure/Work pressure					⊕	⊕			
7, 12	Level of job speed/Time pressure		⊕			+				
11	Facilitation of physical tasks	Manual work				−				
11, 15	Content, scope and amount of routine tasks					−				−
14	Physical demands					+				
11	Diagnosing and navigating demand	Mental work				+				
6, 11	Monitoring tasks/demand					⊘+				
6, 13	Problem-solving demand					⊘+				
15	New challenging mental tasks									+
2	Invasion of privacy	Privacy		⊕⊖⊕						
9, 16	Managerial- or peer surveillance				/		+			
10	Control over work-related data								−	
10	Managerial tracking of behavior								+	
1, 6, 21	Method-order autonomy/Time method control	Autonomy	⊕⊖	+		⊖				
3, 6	Job decision latitude/Job enrichment			⊕⊕⊖⊘		⊘				
7	Job autonomy		⊘							
5, 8	Autonomy					⊖	⊕			
17, 18, 21	Flexibility			+++						
2	Role ambiguity	Role expectations		⊕⊕⊘						
6, 14, 19	Role expansion					⊘+−				−
21, 16	Availability, connectivity and networking pressure			++			+			
9, 20	Meaningful content of work				−	+−				
4, 9	Complexity	Complexity			⊕⊕			+		
7	Job complexity		⊘							
12	Situational awareness					−				
9, 19, 20	Opportunities for learning, and professional development	Opportunities for development				+−		+		−
12, 17	Continuous qualification demands			+		+				

*+, positive relationship; –, negative relationship; /, explicitly no relationship; Quantitative results are encircled; the total number of effects may exceed the number of studies reported due some studies reporting several different effects*.

Subsequently, the results shown are now used as a basis for the identification of work demands that lead to the need for adapting to changes in work characteristics.

### Relationships Between Technologies and Work Demands

Three sources are considered for the identification of work demands: Work demands mentioned in the studies on technology and work characteristics, work demands mentioned by the supplementary studies found during the database search (*N* = 7), and work demands analytically derived from the results.

Some studies that examined the effects of technology on work characteristics also reported concrete work demands. Regarding the increasing complexity and the associated mental work, qualitative evidence suggests an increasing demand for cognitive as well as digital skills (11) in automated systems. With regard to IT systems, quantitative evidence indicates positive relationships with computer literacy (9), and analytical skills (4). With regard to the increase in workflow interruptions and the role expectations for constant availability and connectivity, time and attention management strategies are proposed in order to cope with the intrusive features of technology (2). Other strategies mentioned in the studies include self-discipline for disengaging from the ubiquitous availability resulting from mobile communication devices (18, 8) as well as the need for reflecting on individual responsiveness when working overtime due to self-imposed pressure to be available at all times (18, 21). Concerning opportunities for development, the willingness and ability to learn and adapt to technological changes and the associated changes in work (15, 4, 12) is emphasized. Moreover, employability is facilitated by using technological tools for professional networking (16).

The supplementary studies provide evidence on the direct relationships between technologies and work demands without the mediating consideration of work characteristics. This evidence is listed in Table 7.

**Table 7:** Supplementary studies on the relationship between technology and work-related demands.

		**Relationship between technological aspects and work-related demands**			**Methodology**		
	**Technology under study**	**Perception of technology**	**Work-related demand**	**Correlation/Effect**	**Study design**	***N* (Domain)**	**References**
22	Computer	Perception of controllability (IV)	Exploratory use (low enrichment environment) (ME)	*r* = 0.37^**^	1, a, i	*N* = 158 (Manufacturing, service, government organizations)	Ghani and Deshpande, 1994
			Exploratory use (high enrichment environment) (ME)	*r* = 0.66^**^			
23	Care robots	Negative impact on employment (IV)	Readiness for robotization (DV)	*r* = −0.40^**^	1, a, i	*N* = 3800 (Home- and healthcare; Finland)	Turja et al., 2019
		Experience in robot use (IV)	Readiness for robotization	*r = 0.1*0^**^			
24	Robots	Daily internet use at work (IV)	Individual level robot acceptance at work (DV)	*B* = 0.40^**^	2, a, i	*N* = 53543 (various domains; Europe)	Turja and Oksanen, 2019
		Robot experience (IV)	Individual level robot acceptance at work	*B* = 1.20^**^			
25	Telemedicine technology	Perceived usefulness (IV) Perceived ease of use (IV)	Attitude (ME) Perceived Technology Control (ME)	*PC* = 0.45^**^ *PC* = 0.11^*^	1, a, i	*N* = 408 (Healthcare; Hong Kong)	Chau and Hu, 2002
26	Blockchain technology	Perceived usefulness (IV) Perceived ease of use (IV)	Attitude (ME) Attitude (ME)	*PC* = 0.86^**^ *PC* = −0.01	1, a, i	*N* = 181 (supply chain management; India)	Kamble et al., 2019
27	Information technology system	Perceived usefulness (IV) Perceived ease of use (IV)	Attitude (DV) Attitude	*r = 0.6*4^**^ *r = 0.4*3^**^	1, a, i	*N* = 204 (Nursing; Hong Kong)	Chow et al., 2012
28	Various workplace technologies	Information demands (IV)	Cognitive skills (DV)	*r = 0.4*7^**^	1, a, i	*N* = 184 (various domains; New Zealand)	Spell, 2001
			Interpersonal skills (DV)	*r = 0.4*0^**^			
			Psychomotor skills (DV)	*r =* −0.13			
		Programmability (IV)	Cognitive skills	*r = 0.0*8			
			Interpersonal skills	*r = 0.1*8^*^			
			Psychomotor skills	*r =* −0.02			
		Number of exceptions (IV)	Cognitive skills	*r = 0.3*2^**^			
			Interpersonal skills	*r = 0.3*1^**^			
			Psychomotor skills	*r =* −0.15			

*IV, independent variable; ME, mediating variable; DV, dependent variable; CV, control variable; r, Pearson's r correlation; B, unstandardized coefficients; Concerning methodology: 1, survey; 2, secondary analysis; a, questionnaires; i, cross-sectional; ii, longitudinal/multiple measurements*;

* *p < 0.05*;

** *p < 0.01*.

There is quantitative evidence for positive relationships between the perception of controllability and exploratory use of computers (22), first-hand experience with robots and readiness for robotization (23, 24), and perceived usefulness and positive attitudes toward telemedicine technology (25), blockchain technology (26), and IT systems in general (27). Further quantitative evidence indicates mixed effects of perceived ease of use. Evidence indicates a positive relationship between perceived ease of use and perceived technological control with regard to telemedicine (25), no relationship between ease of use and attitude regarding blockchain technology (26), and a positive relationship between ease of use and attitude toward using IT systems (27). Quantitative evidence indicates that information processing enabled by technology is positively related to an increasing demand of cognitive skills (e.g., synthesizing and interpreting data) and interpersonal skills (e.g., coordinating and monitoring other people), but not related to an increasing demand in psychomotor skills (e.g., manual producing and precise assembling) (28). The level of standardization of work is positively related to interpersonal skills, but not related to cognitive and psychomotor skills (28). A high variety of tasks is positively related to the demand for cognitive skills and interpersonal skills and not related to psychomotor skills (28).

By analyzing the evidence on relationships between technology and work characteristics, further work demands can be derived. Knowledge about the specific technology at hand may be useful to decrease the perception of complexity as new technologies are introduced. This seems evident when comparing the effects of a simple computer with the effects of work within an automated system. For instance, while evidence indicates no relationship between computer work and complexity (6), work within an automated system is suggested to be associated with increasing complexity (12). Moreover, problem-solving skills (13) and cognitive skills such as diagnosing and monitoring (11, 15) increase when employees work within automated systems. Increasing autonomy suggests the need for personal skills regarding self-organizing and self-management due to greater flexibility and the associated possibilities for structuring work in many ways, particularly when working with ICTs (18, 21). Workflow interruptions and an increasing workload also increases the importance of communication skills for explicating the boundaries of one's own engagement to colleagues and leaders (17, 18, 21). Furthermore, reflecting the professional role at work may be critical due to changes in role expectations. The example of self-imposed need for availability underlines this argument (21). All this has implications for self-regulatory activities, such as reflection, and could benefit from experimenting and monitoring one's own strategies for time and attention management.

### Implications for CVET: Objectives and Characteristics

The aforementioned studies describe several required behavioral aspects that are considered important due to technology at work. Emphasized is the need for components related to the organization of one's own work, namely self-discipline and time and attention management.

The identified need for reflection on one's own professional actions, for experimentation, and also for professional networking (for instance by using tools) can be seen as parts of further professional development by oneself or in interaction with others. In addition, the need for demonstrating employability is mentioned. From all these professional and career development aspects can be derived that problem-solving skills, self-regulation skills, and communication skills are required as well as proactive work behavior and coping and reflection strategies.

Various relevant skills, such as psychomotor skills, analytical skills, management skills, and interpersonal skills are mentioned. In addition, the need for diagnostic and monitoring skills as well as digital skills is emphasized. All these components can be used in relation to two explicitly mentioned needs: ability to learn and computer literacy. The demand for generic and transferable skills is emphasized. As a basis for the skills, knowledge is required, for instance on the technology itself, although not explicitly discussed in the studies. In contrast, several components of attitude are explicitly mentioned and considered to be a requirement for the ability to deal with challenges caused by new technologies at work. Firstly, the more generic willingness to learn, adaptability, and perceived behavioral control. Secondly, attitudes that are directly linked to technology, namely a positive attitude and trust, especially toward technology (e.g., robots), and technological readiness and acceptance.

Next to the opportunity of acquiring the mentioned components of competences at work, CVET can organize training interventions in the form of adequate learning environments to foster these. The ability of employees to carry out, develop and use the mentioned behavioral aspects, skills, knowledge, and attitudes, can be considered as required objectives of CVET and have concrete consequences for the characteristics of the learning environments.

As for the content of the learning environments, derived from the aforementioned requirements, it can be argued that attention should be paid to different categories of learning objectives: acquiring knowledge about and learning how to use technology, how to manage work and oneself, and how to continue one's own professional development. In addition, the relevance of attitude tells us that these components need to be fostered in the training and therefore need to be part of the content of the learning environments as well.

In relation to the methods or the didactics, only one study explicitly mentioned a suggestion, namely experience based learning for fostering adaptability (12). In relation to the guidance of trainers or teachers no suggestions are provided. The same goes for assessment, diagnoses or monitoring, and the coherence of components of the learning environments.

## Discussion

This systematic literature review aimed at identifying effects of new technological developments on work characteristics, identifying associated work demands, and determining their implications for the design of formal CVET learning environments.

### Effects of New Technologies on Work Characteristics and Word Demands

Based on a systematic review focusing on empirical evidence, several effects of technology on work characteristics were found, thus answering RQ 1. Evidence suggests that complexity and mental work increases with ongoing automation and robotization of work, for instance due to the automatization of procedures which “hides” certain processes from employees. The automatization of tasks introduces new mental tasks, such as monitoring the machine's activities and solving problems. A decrease in manual work depends on the relation between the job and the technology in use (supporting vs. being supported).

Workload and workflow interruptions increase as a general consequence of the ubiquity of technology, mainly due to a higher level of job speed and the associated time and workload pressure. A higher level of autonomy seems to be associated with a higher workload and more workflow interruptions. This applies in particular to work with ICTs and domain-specific technologies, such as field technology.

Role expectations and opportunities for development depend on the relation between the job and the technology in use (supporting vs. being supported). With regard to role expectations, the need for being available or connected via digital devices and a new division of responsibilities between employees and technology are repeatedly mentioned in the studies. This applies particularly to work with automated systems, robots, and domain-specific technologies such as clinical technology.

With regard to work demands, employees need strategies to deal with higher levels of workload, autonomy, and complexity. Required skill demands contain mental, analytical, cognitive, and self-regulatory demands. In addition, opportunities for role expansion and learning, which do not seem to automatically result from the implementation and use of new technologies, need to be created (pro)actively by the employees. Employees need to take more responsibility with regard to their own development and professional work identity (for instance considering the pressure for constant availability). They need to be able to effectively deal with a high workload and number of interruptions, increasing flexibility, complexity, and autonomy, a demand for constant availability, changes in meaningfulness of tasks, changes in work roles, and the need to create and use learning opportunities. In the light of ongoing changes and challenges, skills to further develop and adapt one's own skills gain in importance. Regarding attitudes, the willingness to learn, adapt and experiment may be a central work demand.

### Implications for the Practice of CVET

Various required objectives of CVET can be concluded from the reported results. For instance, developing the ability of employees to carry out the mentioned behaviors, as well as the skills, knowledge and attitudes that are necessary for those behaviors. These objectives have consequences for the content of CVET learning environments. From the empirical studies on the relationships between technology and work, we derived the need for employees to organize their own work, for instance through time management. Furthermore, many issues relating to own professional development and career development are important, to acquire individually and independently as well as by interacting with others. Ultimately, this refers to the skills of self-initiated learning and development. With regard to fostering helpful attitudes, raising awareness of the relevance of trust or training the social skills to promote trust in the workplace can be included in the content of CVET learning environments. In research on creating trust within organizations, regularly giving and receiving relevant information was shown to be important for creating trust toward co-workers, supervisors and top-management, which in turn fostered the perception of organizational openness and employee involvement as a result (Thomas et al., 2009). In the research on creating trust in virtual teams, the importance of frequent interaction was important to develop trust on a cognitive as well as an affective level (e.g., Germain, 2011). These research results however need to be adapted to the context of technology at work.

Although there is no information provided on the guidance of employees, informal guidance through leadership (Bass and Avolio, 1994) as well as formal guidance by trainers and teachers during interventions contain possibilities for fostering the required competences. Attention should be paid not only to acquiring relevant knowledge (digital literacy), but also to skills in applying the knowledge and therefore dealing with technology. Even more challenging might be the task of supporting attitude development (e.g., technological acceptance and openness to changes), fostering transfer of skills, and preparation for future development. Especially future professional development, which includes the ability to learn in relation to current and future changes, needs to be focused on. Teachers, trainers and mentors need to be equipped to be able to foster these competences.

In relation to the use of didactical methods, methods that do not merely focus on knowledge acquisition but also provide opportunities for skill acquisition and changes in attitude need to be applied. For example, one study explicitly suggested experience based learning for fostering the adaptability of employees when faced with ongoing technological developments. Other solutions for instruction models as a profound basis for learning environments may be found in more flexible approaches, for instance according to the cognitive flexibility theory (Spiro et al., 2003), where learners are meant to find their own learning paths in ill-structured domains. By applying such models, that are often based on constructivist learning theories, in a coherent way, the development of strategies for self-organizing and self-regulation may be facilitated.

Furthermore, the use of technology within learning environments may have the potential to increase participants interactions, which are focused in for instance collaborative and co-operative learning (Dillenbourg et al., 2009). Next to increasing interactions in learning and being able to co-operate, technology in learning environments can used to foster the other required competences, if adequately designed (Vosniadou et al., 1996; Littlejohn and Margaryan, 2014).

When keeping in mind, that the coherence of components is an important requirement for the design of learning environments (Mulder et al., 2015), the component that describes assessment needs further attention. There is evidence supporting the idea, that the type of assessment has an impact on how learning takes place (Gulikers et al., 2004; Dolmans et al., 2005). Therefore, it can be used to deliberatively support and direct learning processes.

Only when all these aspects are considered can CVET interventions effectively and sustainably foster the mentioned objectives, such as promoting a willingness to change in relation to technologies, the effective use of technology, and personal development in the context of technological developments.

### Limitations and Implications for Future Research

Regarding the search methods, the use of databases is challenging when investigating technologies (Huang et al., 2015). Technological and technical terms are widespread outside the research in which they are regarded as the object of investigation. Therefore, it produces a large amount of studies that concern technology with diverse research objectives that can be difficult to sort. An interesting focus for future research would be the systematic mapping of journals dealing specifically with technology in order to identify research that could complement the results of the present study as well as consider specificities regarding the domains in which the data is collected and disciplines by which the research is conducted. For instance, domain-specific databases from healthcare or manufacturing might provide additional insights into the effects of technology on work. Another limitation is the absence of innovative new technologies, such as artificial intelligence, blockchain, or the internet of things as object of investigation. Broad technological categories, such as ICTs and social media have received some attention in research, especially in relation to questions beyond the scope of this review. Newer technological developments as discussed by Ghobakhloo (2018) are virtually not present in current research. This gap in empirical research needs to be filled. In addition, future research should ensure that it does not miss opportunities for research where effects of these innovative technologies can be examined in detail, for instance by conducting an accompanying case study of the implementation process. Research investigating changes over time regarding the use of technology and its effects is needed. In doing so, research could capture the actual dynamics of change and development of processes as they happen in order to inform truly effective interventions in practice. Moreover, a classification of technological characteristics according to their effects may be valuable by enabling a more in-depth analysis of new technologies and their effects on specific groups of employees and different types of organizations. These analyses will also allow a breakdown of effects in relation to differences in jobs, hierarchy levels and levels of qualification, which could be very important for organizations and employers in order to adapt the CVET strategy to the specific demands of specific groups of employees. The present review takes a first step in this direction by identifying work characteristics that are affected by different technologies. In addition, future research could also take into account non-English language research, which might increase insight in for instance cultural differences in the use and the effects of technology at work.

Regarding theory, some of the relevant theories considering technology stem from sociology (e.g., Braverman, 1998) or economics (Autor et al., 2003). For instance, the task-based approach (Autor et al., 2003) showed some explanatory value by suggesting that complexity may increase as a consequence of technology. Furthermore, it suggested that this effect may depend on job specifics. Those propositions are reflected in the aforementioned empirical evidence. Psychological theories on work characteristics do not conceptualize technology explicitly (e.g., Hackman and Oldham, 1975; Karasek, 1979). As of the present study, the large variation regarding the concepts and variables derived from theory might limit the comparability of results. To foster systematic research, further theory development needs to more explicitly consider the role of technology at multiple levels (i.e., individual level, team level, organizational level) and with regard to the characteristics and demands of work. In the context of theory, the paradigmatic views also deserve attention (e.g., Liker et al., 1999; Orlikowski and Scott, 2008). These views could be reflected in the subject of research, as exemplified for instance in the study of field technologies and its effects on privacy from a managerial control and power perspective, potentially reflecting the view of political interest (Tranvik and Bråten, 2017). Most of the studies, however, do not take a clear stand on what exactly they mean when they investigate technology. This complicates interdisciplinary inquiry and integration, as it is not always clear which understanding of technology is prevalent. We therefore encourage future research to explicitly define technology, for instance as in the present paper using the proposed framework of McOmber (1999). In doing so, characteristics of technology may be defined more clearly and distinctive which in turn would enable the formation of the strongly needed categorization of technologies, as was proposed earlier.

And, although there are theories and models on the use of technology in education (e.g., E-Learning, Technology enhanced learning), they are not focussing on fostering the competences required to deal with new technologies in a sustainable manner. In general, the same gap needs to be filled for instruction models and instructional design models, for instance to promote changes in attitude and professional development. In addition, there is hardly any attention for the consequences of new technologies at work for CVET yet (Harteis, 2017). All this requires more systematic evaluation studies. The research gaps identified need to be filled in order to provide evidence-based support to employees in dealing with new technologies at work in a sustainable manner, taking charge of their own performance and health, as well as seeking and using opportunities for their own professional and career development.

## Data Availability Statement

All datasets generated for this study are included in the article/supplementary material.

## Author Contributions

PB and RM have jointly developed the article, and to a greater or lesser extent both have participated in all parts of the study (design, development of the theoretical framework, search, analyses, and writing). The authors approved this version and take full responsibility for the originality of the research.

## Conflict of Interest

The authors declare that the research was conducted in the absence of any commercial or financial relationships that could be construed as a potential conflict of interest.
